# Assessment of fractional flow reserve in intermediate coronary stenosis using optical coherence tomography-based machine learning

**DOI:** 10.3389/fcvm.2023.1082214

**Published:** 2023-01-25

**Authors:** Jung-Joon Cha, Ngoc-Luu Nguyen, Cong Tran, Won-Yong Shin, Seul-Gee Lee, Yong-Joon Lee, Seung-Jun Lee, Sung-Jin Hong, Chul-Min Ahn, Byeong-Keuk Kim, Young-Guk Ko, Donghoon Choi, Myeong-Ki Hong, Yangsoo Jang, Jinyong Ha, Jung-Sun Kim

**Affiliations:** ^1^Division of Cardiology, Cardiovascular Center, Korea University Anam Hospital, Korea University College of Medicine, Seoul, Republic of Korea; ^2^Department of Electrical Engineering, Sejong University, Seoul, Republic of Korea; ^3^Faculty of Information Technology, Posts and Telecommunications Institute of Technology, Hanoi, Vietnam; ^4^School of Mathematics and Computing (Computational Science and Engineering), Yonsei University, Seoul, Republic of Korea; ^5^Yonsei Cardiovascular Research Institute, Yonsei University College of Medicine, Seoul, Republic of Korea; ^6^Division of Cardiology, Severance Hospital, Yonsei University College of Medicine, Seoul, Republic of Korea; ^7^Division of Cardiology, CHA Bundang Medical Center, CHA University College of Medicine, Seongnam, Republic of Korea

**Keywords:** machine learning, fractional flow reserve, optical coherence tomography, preoperative planning, cardiovascular imaging

## Abstract

**Objectives:**

This study aimed to evaluate and compare the diagnostic accuracy of machine learning (ML)- fractional flow reserve (FFR) based on optical coherence tomography (OCT) with wire-based FFR irrespective of the coronary territory.

**Background:**

ML techniques for assessing hemodynamics features including FFR in coronary artery disease have been developed based on various imaging modalities. However, there is no study using OCT-based ML models for all coronary artery territories.

**Methods:**

OCT and FFR data were obtained for 356 individual coronary lesions in 130 patients. The training and testing groups were divided in a ratio of 4:1. The ML-FFR was derived for the testing group and compared with the wire-based FFR in terms of the diagnosis of ischemia (FFR ≤ 0.80).

**Results:**

The mean age of the subjects was 62.6 years. The numbers of the left anterior descending, left circumflex, and right coronary arteries were 130 (36.5%), 110 (30.9%), and 116 (32.6%), respectively. Using seven major features, the ML-FFR showed strong correlation (*r* = 0.8782, *P* < 0.001) with the wire-based FFR. The ML-FFR predicted wire-based FFR ≤ 0.80 in the test set with sensitivity of 98.3%, specificity of 61.5%, and overall accuracy of 91.7% (area under the curve: 0.948). External validation showed good correlation (*r* = 0.7884, *P* < 0.001) and accuracy of 83.2% (area under the curve: 0.912).

**Conclusion:**

OCT-based ML-FFR showed good diagnostic performance in predicting FFR irrespective of the coronary territory. Because the study was a small-size study, the results should be warranted the performance in further large-scale research.

## 1. Introduction

Fractional flow reserve (FFR) is the gold standard strategy for decision-making for revascularization therapy in patients with intermediate coronary artery lesions (1, 2). In spite of the improved evidence of FFR-guided percutaneous coronary intervention (PCI), several concerns, including the need for drug-induced hyperemia and prolonged procedure time, may limit the use of FFR in clinical practice (3). Optical coherence tomography (OCT) is a high-resolution imaging modality for planning PCI because it can provide several information on the exact characteristics of lesions (4). However, the association between lesion characteristics and hemodynamic significance has not been fully elucidated owing to its high cost (5). To reduce the theoretical gap between lesion characteristics and hemodynamic significance, a computational flow dynamics simulation was reported to predict hemodynamics based on OCT images, and this showed a good correlation with clinical FFR. However, such a simulation requires a complicated time-consuming process, which is a limitation to its use in clinical practice (6). Machine learning (ML) techniques have been widely used for fast and effective analysis of the association between lesion characteristics and hemodynamics in coronary artery disease, based on the identification of patterns in large datasets with a multitude of variables (7–9). Recently, ML techniques for predicting the hemodynamic significance of the left anterior descending artery (LAD) in terms of FFR using OCT images and biometric information have been reported (10). However, there has been no study on a global OCT-based ML model to predict FFR in all coronary artery territories. Thus, the present study aimed to evaluate and compare the diagnostic accuracy of ML-FFR based on OCT with wire-based FFR, irrespective of the coronary territory.

## 2. Materials and methods

The Integrated Coronary Multicenter Imaging Registry was conducted to investigate the clinical association of intra-coronary OCT and the FFR in patients with intermediate coronary stenosis, which was collaborated between four institutions in South Korea (ClinicalTrials.gov, Identifier: NCT03298282). Detailed information was reported previously (11). Briefly, a total of 180 patients enrolled the registry and performed both pressure wire-based FFR measurement and OCT examination for all coronary territory with intermediate stenosis (40–70%). After the exclusion due to no intermediate stenosis, poor image quality of OCT, and incomplete OCT coverage of the entire lesion, a total of 356 coronary arteries from 130 patients with intermediate stenosis were included in the analysis. And pressure wire-based FFR was used as a reference to assess the diagnostic performance of OCT-based ML-FFRs. The inclusion criteria were as follows: (1) patients who underwent coronary CT angiography because of chest pain; and (2) a de novo lesion of intermediate stenosis (diameter stenosis = 40–70%) in the proximal to the middle portions. The exclusion criteria were: (1) patients presenting myocardial infarction with single vessel disease; (2) hypersensitivity to the contrast agent; (3) use of inotropic agents owing to hemodynamic instability; (4) severe ventricular dysfunction (left ventricular ejection rate < 30%); (5) creatinine levels ≥ 2.0 mg/dL, (6) life expectancy of < 12 months owing to noncardiac comorbidity; and (7) severe heart valve disease. This study was approved by the institutional review board of Severance Hospital, and complied with the principles of the Declaration of Helsinki. Written informed consent was obtained from all the patients.

### 2.1. OCT measurements

A frequency-domain OCT system (C7-XR OCT imaging system; LightLab Imaging, Inc., Abbott Vascular, Chicago, IL, USA) was used to acquire OCT images with the same method as a previous report (10). At a rate of 100 fps, Cross-sectional OCT images were obtained with 20mm/s of pullback velocity. The core laboratory (Cardiovascular Research Center) analyzed the OCT data without patients and procedural information. The minimal luminal area (LA) was defined as the segment with the smallest LA on OCT analysis. The proximal and distal reference LA were within the same segment as the lesion with the largest lumen. Both reference LA were usually within 10 mm of the stenosis, without major intervening branches (12). The minimal LA used to define functional stenosis according to the OCT criteria was 1.96 mm (6, 13). The percentage of stenotic area (%) was defined as [(mean reference LA - minimum LA)/mean reference LA] × 100. The method of OCT analysis in the core laboratory were as following; manual contour delineation, measure minimal lumen area with 1mm interval analysis, and set value at certain point on each proximal, distal reference lumen area. In this study, OCT analysis of the lesions and a detailed explanation of the analyzed features were based on previous OCT studies (14, 15).

### 2.2. Wire-based FFR measurements

Wire-based FFR measurement was conducted in a usual manner with a 0.014-inch pressure guidewire (Abbott Vascular, Chicago, IL, USA) during coronary angiography. After equalization on the coronary ostium, the pressure guidewire was set distal to the target lesion. After the wire was on set, continuous infusion of intravenous adenosine (140 μg/kg/min) via the antecubital vein was done for acquiring maximal hyperemia. The FFR was calculated in terms of mean hyperemic distal coronary pressure/mean aortic pressure. FFR ≤ 0.8 was defined as functionally significant stenosis. A pressure drift of ± 3 mmHg was considered acceptable. If the pressure drift exceeded this margin, repeated FFR recording was performed.

### 2.3. ML-FFR assessment based on OCT

Within the broad field of ML, supervised learning regression has been utilized for many applications in predicting FFR (7, 16). In a regression problem, a model is supervised to establish a relationship among variables by estimating how one variable affects the other. Regression was performed according to the following steps: (1) feature selection; (2) choosing an ML algorithm; (3) training the model; (4) evaluating the model; and (5) parameter tuning. In this study, a random forest (RF) was used to estimate the FFR. RF is an ensemble of decision trees created on a subset of data. The regression value is the combined output of all decision trees. In this way, it not only prevents overfitting but also reduces variance; thus, accuracy is improved, even on an imbalanced dataset. It is worth noting that deep learning-based methods, while recently gaining considerable attention, are not a good choice for the FFR regression problem because of the lack of training data; thus, deep learning-based methods are prone to overfitting. Feature selection was performed as previously described (10). In brief, the features for developing an ML-FFR model of coronary intermediate lesions were selected in terms of clinical characteristics or OCT findings according to expert opinion by worldwide guidelines and a prior literature search (14, 15, 17, 18).

Including the aforementioned 36 features, we used six key features from OCT image analysis and one feature representing the vessel type by computing the correlation coefficients between the features and clinical FFR values. The training and testing groups were divided into three groups, each containing vessel type [LAD, left circumflex artery (LCx), and right coronary arteries (RCA)] by a stratified sampling technique (19), in order to prevent leaving out a sub-group and leading to sampling bias (20). In the training phase, the model was independently trained on the 356 datasets with a ratio of training: testing = 4:1 and the top seven extracted features (OCT features and type of vessel). To prevent instability and optimize the performance of the model, cross validation (CV) and hyperparameter tuning (GridSearchCV) were performed on the training set data. The training set was split into K subsets, which are also known as folds. In our study, we fitted a RF model with K = 5. A RF approach was performed using many iterations of the entire 5-folds CV process, each time using different combinations of hyperparameters. The optimal values for the hyperparameters of RF algorithm for the FFR regression problem are summarized in Supplementary Table 1 (*n_estimators = 64, max_depth = 16, max_features = 3, min_sample_leaf = 3 and min_samples_split = 12*), and the default values of the other remaining parameters were utilized. Specifically, the optimized parameter values were achieved after 1080 combinations of settings and consumed 1.1 min (Windows 10, Intel^®^ Core™ i7-7770 CPU at 3.60 Hz (eight CPUs), 24Gb RAM, NVIDIA GeForce GTX 1060 6GB). Using the optimized parameters, a RF model was established. In the testing phase, 72 coronary arteries with seven key features were tested online using trained models to predict the FFR. Pearson correlation coefficient and mean absolute error (MAE) were used to evaluate the RF model.

### 2.4. External validation

To investigate the performance of the developed ML model, the ML model was validated by external data, which is one hundred one intermediate coronary artery stenosis of the forty-seven patients from Integrated Coronary Multicenter Imaging Registry – Extended (ClinicalTrials.gov, Identifier: NCT04153903).

### 2.5. Statistical analyses

Continuous variables were analyzed with descriptive methods depending on distribution; mean ± standard deviations or medians. Categorical variables were analyzed as numbers with percentages. Taking considering the normality of each quantitative variable, Student’s *t*-test or Mann- Whitney test was performed for continuous variables. To evaluate the relationships between pressure wire-based FFR and computational FFRs (Fusion-FFR, CT-FFR, and OCT-FFR), Pearson correlation coefficient analysis and the Bland–Altman analysis were performed. The diagnostic performance of the OCT-based ML-FFR in assessing functionally significant stenosis was checked by the Receiver operating characteristics (ROC) curve analysis. The sensitivity analysis of OCT-based ML-FFR regarding accuracy, sensitivity, specificity, positive predictive value, and negative predictive values were calculated. Statistical analyses were performed using IBM SPSS, version 25.0 (IBM Corp., Armonk, NY, United States), and MedCalc, version 20.110 (MedCalc Software, Ostend, Belgium). All tests were two-sided, and a *P*-value < 0.05 was considered statistically significant.

## 3. Results

### 3.1. Clinical and lesion characteristics

The mean age of the subjects was 62.5 years. Approximately 74.6% of the study population were men. Hypertension and diabetes mellitus were diagnosed in 60.8 and 31.5% of subjects, respectively. A total of 29.2% of the patients presented with acute coronary syndrome. The numbers of the LAD, LCx, and RCA were 130 (36.5%), 110 (30.9%), and 116 (32.6%), respectively. A median value of invasive FFR of the derivation cohort was 0.92 (0.83–0.97) regardless of coronary artery territory [LAD, 0.83 (0.77–0.87); LCx, 0.95 (0.90–0.98); RCA, 0.96 (0.92–0.98)] (Table 1). And the median values of invasive FFR for training, testing, and external validation group were 0.92, 0.93, and 0.88, respectively (Supplementary Table 2). Fractional flow reserve of A positive result ratio of invasive FFR (FFR ≤ 0.8) of the training set, the testing set, and the external validation set was 20.1, 18.1, and 33.7%, respectively.

**TABLE 1:** Baseline characteristics of 130 patients with 356 coronary lesions.

Clinical data	
Age (years)	62.5 ± 8.8
Sex, male, *n* (%)	97 (74.6)
Coronary artery location, *n* (%)	
- Left anterior descending	130 (36.5)
- Left circumflex	110 (30.9)
- Right coronary artery	116 (32.6)
Systolic blood pressure (mmHg)	131.9 ± 19.3
Diastolic blood pressure (mmHg)	75.3 ± 11.0
Height (cm)	165.9 ± 8.0
Weight (kg)	69.8 ± 10.4
Body mass index (kg/m^2^)	25.3 ± 3.0
Acute coronary syndrome, *n* (%)	38 (29.2%)
Hypertension, *n* (%)	79 (60.8%)
Diabetes mellitus, *n* (%)	41 (31.5%)
Hypercholesterolemia	58 (44.6%)
Current smoking, *n* (%)	29 (22.3)
Pre-procedural hemoglobin level (mg/dL)	14.3 ± 1.3
Pre-procedural platelet count (10^3^ μL)	234.7 ± 60.8
Pre-procedural BUN level (mg/dL)	15.8 ± 4.4
Pre-procedural creatinine level (mg/dL)	0.8 ± 0.1
Fractional Flow Reserve (*n* = 356)	0.92 (0.83–0.97)
- Left anterior descending (*n* = 130)	0.83 (0.77–0.87)
- Left circumflex (*n* = 110)	0.95 (0.90–0.98)
- Right coronary artery (*n* = 116)	0.96 (0.92–0.98)
**Optical coherence tomography parameters**	
Proximal lumen area (mm)	7.6 ± 3.4
Minimal lumen area (mm)	3.6 ± 2.5
Distal lumen area (mm)	8.2 ± 3.6
Lesion length (mm)	22.7 ± 12.0
Plaque area	14.6 ± 5.0
Area stenosis (%)	76.4 ± 11.0
Calcified nodule	34 (9.6)
Lipid-rich plaque, *n* (%)	87 (24.4)
Lipid arc over 90 degrees, *n* (%)	60 (16.9)
Lipid arc over 90 degrees with thickness < 65 μm, *n* (%)	24 (6.7)
Existence of dissection, *n* (%)	16 (4.5)
Existence of necrotic core, *n* (%)	162 (45.5)
Existence of microvessels, *n* (%)	94 (26.4)
Existence of cholesterol crystal, *n* (%)	137 (38.5)
Existence of rupture, *n* (%)	42 (11.8)
Existence of erosion, *n* (%)	24 (6.7)
Existence of macrophage, *n* (%)	34 (9.6)

BUN, blood urea nitrogen.

### 3.2. Major features of the OCT-based ML-FFR and its performance

**FIGURE 1:**
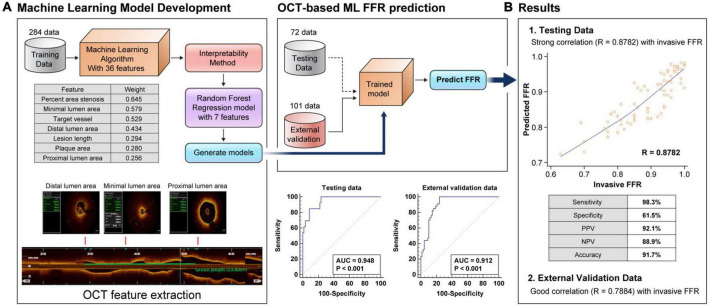
Flow chart of the OCT-based machine learning method and weight of each feature **(A)**. Comparison between the clinical fractional flow reserve results and the predicted fractional flow reserve results in the random forest model in the testing set and external validation set, and receiver operating characteristic curve of machine learning-fractional flow reserve **(B)**. FFR, fractional flow reserve; AUC, area under the curve.

The present study developed an OCT-based ML-FFR using a RF model that included seven major features. The seven major features were selected from a total of 36 features based on weight as follows: percent area stenosis, vessel type, minimal LA, lesion length, distal LA, proximal LA, and plaque area (Figure 1).

Figure 1 illustrates the predicted results of the RF model using the seven most important features compared with the clinical FFR of the testing set. The results showed a good correlation (*r* = 0.8782, *P* < 0.001) and agreement (MAE = 0.0344) between the OCT-based ML-FFR and wire-based FFR. In the analysis of the Bland-Altman plot, the statistical limits of the OCT-based ML-FFR were 0.01 ± 0.09, based on the wire-based FFR (Supplementary Figure 1). Based on an FFR ≤ 0.8, the sensitivity, specificity, positive predictive value, negative predictive value, and accuracy of the OCT-based ML-FFR method in the testing group were 98.3, 61.5, 92.1, 88.9, and 91.7%, respectively. To assess whether the present OCT-based ML-FFR was accurate, external validation was conducted using a total of 101 coronary arteries. The results showed a good correlation (*r* = 0.7884, *P* < 0.001) between ML-FFR and wire-based FFR(Supplementary Figure 2). Based on an FFR ≤ 0.8, the sensitivity, specificity, positive predictive value, negative predictive value, and accuracy of the OCT-based ML-FFR method in the external validation data were 89.6, 70.6, 85.7, 77.4, and 83.2%, respectively (Table 2).

**TABLE 2:** Model performance and sensitivity analysis.

	Testing (*n* = 72)	External validation (*n* = 101)
Pearson correlation	0.8782	0.7884
Sensitivity	98.3%	89.6%
Specificity	61.5%	70.6%
Positive prediction value	92.1%	85.7%
Negative prediction value	88.9%	77.4%
Accuracy	91.7%	83.2%

## 4. Discussion

Machine-learning-based approaches for analyzing associations between numerous variables have been widely addressed to complement and improve existing prediction models. The main findings of the present study that developed OCT-based ML-FFR prediction were as follows: We developed an OCT-based ML algorithm for FFR prediction using the RF method based on clinical and OCT features. The developed model with seven major features showed the best performance in predicting the FFR, with a correlation of 0.87. The seven major features, including the ML model, were as follows percent area stenosis, vessel type, minimal LA, lesion length, distal LA, proximal LA, and plaque area. The sensitivity, specificity, and accuracy of OCT-based ML-FFR were 98.3%, 61.5%, and 91.7%, respectively. To our knowledge, this is the first general model of OCT-based ML-FFR assessment irrespective of the coronary territory.

Fractional flow reserve is the gold standard strategy for ischemia-guided revascularization in current guidelines; moreover, there are evidence of better outcomes in FFR-guided PCI than in angiography-guided PCI (1, 2). However, FFR is still used less in clinical practice and has unsolved questions, such as limitations in reflecting lesion characteristics. Moreover, although the Fractional Flow Reserve versus Angiography for Multivessel Evaluation (FAME) trial reported a reduction in 1-year major adverse cardiac events of FFR-guided PCI in multivessel disease compared to angiography-guided PCI (21), the FAME 3 trial demonstrated that FFR-guided PCI was not inferior to CABG (22). However, in the FAME 3 trial, intravascular image-guided PCI, which may benefit outcomes, was performed in only 11.7% of the total PCI procedures. These observations suggest that FFR alone is insufficient for achieving improved clinical outcomes, and the role of intravascular imaging in determining lesion characteristics may be necessary.

Optical coherence tomography is a useful diagnostic tool before PCI, as it provides several information, including morphological plaque description (fibrous, fibrocalcific, lipid-rich plaque, thin-cap fibroatheroma), accurate dimensional measurement, identification of thrombus, and underlying culprit lesion phenotype (ruptured fibrous cap, intact fibrous cap) (4, 23). Based on this information, OCT-guided PCI may improve clinical outcomes in patients with stable coronary artery disease and acute coronary syndrome (1, 2). Furthermore, a recent single-center randomized study reported that OCT-guided PCI reduced major adverse cardiac events and significant angina compared to FFR-guided PCI (24). However, despite the promising results of OCT, it is still used less in practice than IVUS (25). Plausible explanations suggest that there are barriers to interpreting it owing to the overwhelming amount of OCT imaging data. In addition, several limitations are well known, such as a lower penetration depth compared with IVUS, which makes it difficult to assess plaque volume in the deep layers of the diseased vessel and increases the radiocontrast burden to achieve a proper OCT image (26). Thus, although intravascular imaging has benefits in PCI results, proof of the cost-effectiveness of OCT-guided PCI is essential for using OCT in routine practice. In addition, the plaque characterization on OCT may be less informative due to the lack of physiologic information for PCI indication regarding non-culprit lesions of multivessel disease. In context, the OCT-based ML FFR of the present study may provide a clue to treatment decisions for a non-culprit lesion by acquiring physiological information through a single OCT procedure. That is, the present method had advantages regarding procedure time, invasiveness, and cost of another procedure because wire-based FFR is not required.

In this context, various image-based mathematical models have been developed to assess hemodynamic significance and improve cost-effectiveness (27). In a single-center observational study, OCT-based computational analysis demonstrated a better correlation with conventional FFR than other imaging modalities, such as computational tomography or IVUS (6, 28). These differences could be explained by the superiority of OCT in terms of the actual contour of lumen acquisition compared to CT, angiography, and IVUS imaging (29). However, CFD algorithms require relatively long processing times and have complex interfaces for clinicians to use in routine practice despite their good correlation with FFR (10, 30). In this context, an OCT-based ML algorithm may be an alternative to CFD algorithms in terms of providing hemodynamic significance in a clinician-friendly manner. In addition, the OCT-based ML method can predict the FFR within 2–3 min using major OCT features derived by automatic contour delineation with full frame analysis, whereas the CFD algorithm requires at least 20 min. This suggests that an OCT-based ML algorithm can be implemented in actual practice in real time to determine the appropriate treatment strategy.

**FIGURE 2:**
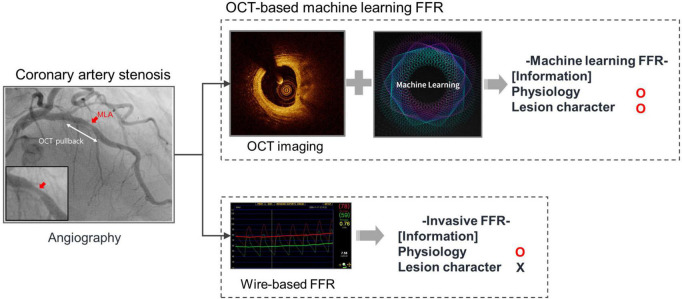
Advantage of OCT-based machine learning FFR.

Several studies have reported the prediction of FFR using ML models based on cardiovascular images, such as CT, coronary angiography, intravascular ultrasound, and OCT (7–10, 31, 32). Although the above studies showed good performance of FFR prediction, there was a tendency to show better performance with respect to high-resolution images. OCT is superior in terms of resolution compared to CT, angiography, and IVUS imaging (26). A recent study reported that OCT-based ML-FFR for the LAD showed 100% sensitivity and 92.9% specificity (10). The present study developed OCT-based ML-FFR, irrespective of the coronary territory, as a global model and showed a good correlation. In addition, it should be noted that high-resolution OCT images can achieve data without bias because OCT image analysis has minimal intra- and inter-observer variability (Figure 2). This suggests that OCT-based ML-FFR may reduce inaccurate feature data input compared with other image modalities.

This study has several limitations. Although a good correlation coefficient was shown between OCT-based ML-FFR and wire-based FFR, this was a small-sized study for patients with intermediate lesions and the sample lesions eventually became highly selective. In addition, compared with the testing group, the correlation coefficients between ML-FFR and wire-based FFR were relatively low in patients in the external validation data. However, this is the first attempt on the OCT-based ML-FFR method irrespective of the coronary territory as well as conducting external validation analysis. Thus, further research is warranted in order to better the performance of ML-FFR methods. In addition, since the present study excluded the side branch information, the impact of the size branch on the OCT-based ML-FFR method should be investigated. Finally, the rationale or clinical impact of decision making using ML methods has not been fully elucidated. However, sufficient information on the target lesion is expected to result in better clinical outcomes after treatment. In addition, although this study was conducted on de novo lesions, further research should investigate of the impact of OCT-based FFR on PCI optimization after OCT-guided PCI and its clinical outcomes. In spite of these limitations, we suggest that OCT-based ML FFR may provide clinicians with important physiological data needed to optimally treat intermediate coronary lesions.

## Data availability statement

The original contributions presented in this study are included in the article/Supplementary material, further inquiries can be directed to the corresponding authors.

## Ethics statement

The studies involving human participants were reviewed and approved by an Institutional Review Board of Severance Hospital. The patients/participants provided their written informed consent to participate in this study.

## Author contributions

All authors listed have made a substantial, direct, and intellectual contribution to the work, and approved it for publication.
