# Supplementary material for: Assessment of fractional flow reserve in intermediate coronary stenosis using optical coherence tomography-based machine learning
Source: Front Cardiovasc Med. 2023 Jan 25;10:1082214. doi: 10.3389/fcvm.2023.1082214 (PMC9905417; doi:10.3389/fcvm.2023.1082214)

Assessment of fractional flow reserve in intermediate coronary stenosis using optical coherence tomography-based machine learning

Supplemental table 1: Page 2

Supplemental figure 1: Page 3

Supplemental Table 1. Random Forest parameters

| Optimized parameters | Description | Value |
| --- | --- | --- |
| n_estimators | Number of trees in Random Forest | 64 |
| max_depth | Maximum number of levels in tree | 16 |
| max_features | Minimum number of samples required to split a node | 3 |
| min_samples_leaf | Minimum number of samples required at each leaf node | 3 |
| min_samples_split | Minimum number of samples required to split an internal node | 12 |

Supplemental Table 2. Fraction flow reserve value regarding the group

| Number of lesions | Training group (n=284) | Testing group (n=72) | External validation group (n=101) |
| --- | --- | --- | --- |
| Fraction Flow Reserve | 0.92 (0.83–0.97) | 0.93(0.83–0.96) | 0.88 (0.77–0.94) |

Supplemental Figure 1. Bland-Altman plot of clinical FFR and OCT-based machine learning FFR


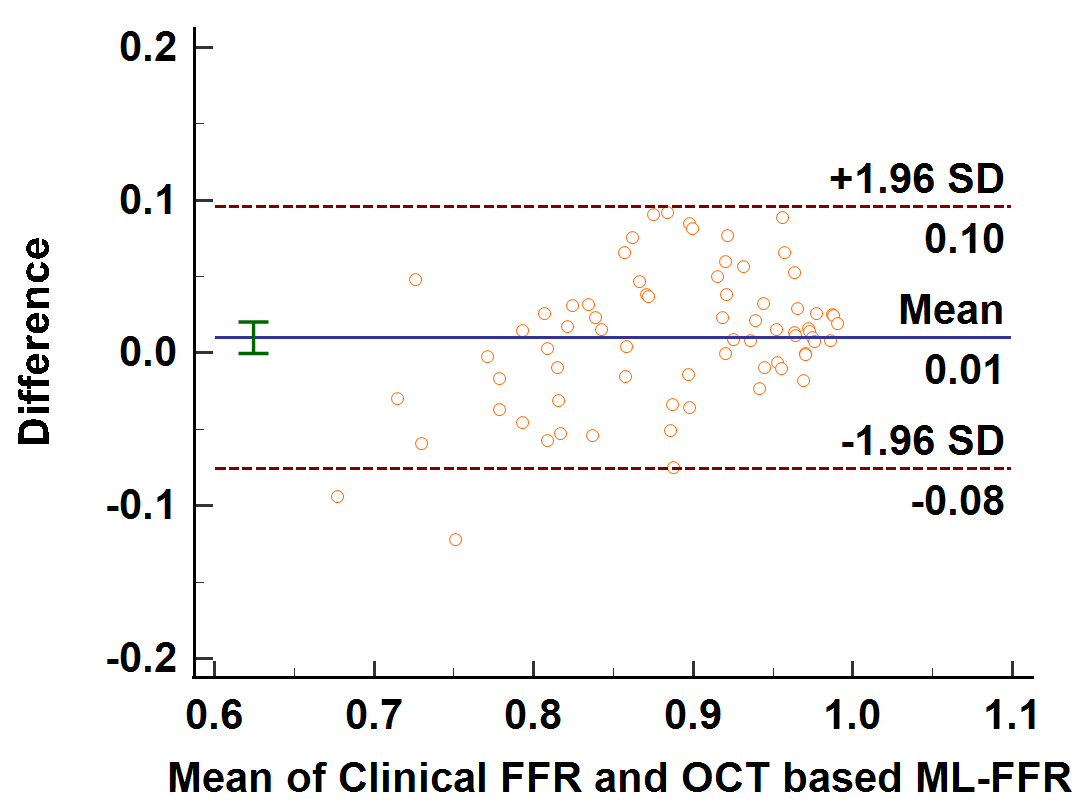


Supplemental Figure 2. Scatter plot in the validation cohort


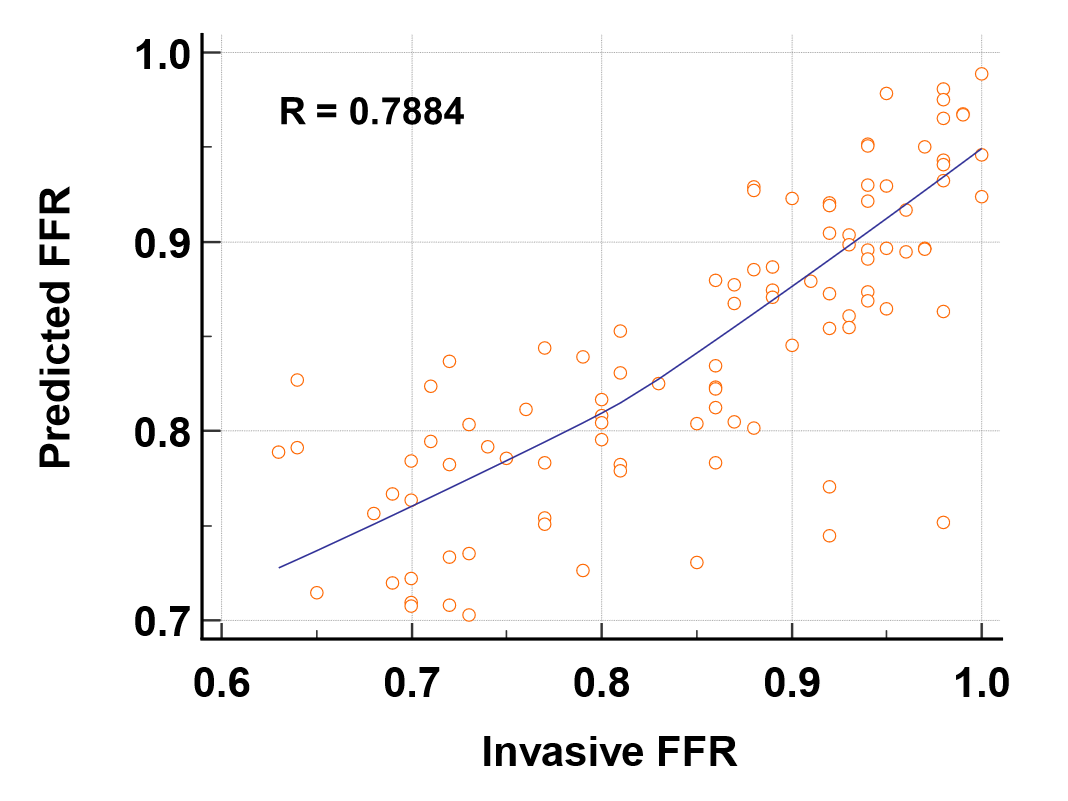

Supplement: Supplementary file 1 [file Table_1.DOCX]
